# High-throughput mammalian two-hybrid screening for protein-protein interactions using transfected cell arrays

**DOI:** 10.1186/1471-2164-9-68

**Published:** 2008-02-06

**Authors:** Andrea Fiebitz, Lajos Nyarsik, Bernard Haendler, Yu-Hui Hu, Florian Wagner, Sabine Thamm, Hans Lehrach, Michal Janitz, Dominique Vanhecke

**Affiliations:** 1 Max Planck Institute for Molecular Genetics, Department Vertebrate Genomics, Fabeckstr. 60-62, 14195 Berlin, Germany; 2 FU Berlin, Department of Biology, Chemistry and Pharmacy, Takustr. 3, 14195 Berlin, Germany; 3 Bayer Schering Pharma, Corporate Research Oncology, Müllerstr. 170-178, 13342 Berlin, Germany; 4 RZPD German Resource Center for Genome Research, Heubnerweg 6, 14059 Berlin, Germany; 5 ATLAS Biolabs, Friedrichstr. 147, 10117 Berlin, Germany; 6 University of Basel, Center for Biomedicine, Mattenstr. 28, 4058 Basel, Switzerland

## Abstract

**Background:**

Most of the biological processes rely on the formation of protein complexes. Investigation of protein-protein interactions (PPI) is therefore essential for understanding of cellular functions. It is advantageous to perform mammalian PPI analysis in mammalian cells because the expressed proteins can then be subjected to essential post-translational modifications. Until now mammalian two-hybrid assays have been performed on individual gene scale. We here describe a new and cost-effective method for the high-throughput detection of protein-protein interactions in mammalian cells that combines the advantages of mammalian two-hybrid systems with those of DNA microarrays.

**Results:**

In this cell array protein-protein interaction assay (CAPPIA), mixtures of bait and prey expression plasmids together with an auto-fluorescent reporter are immobilized on glass slides in defined array formats. Adherent cells that grow on top of the micro-array will become fluorescent only if the expressed proteins interact and subsequently trans-activate the reporter. Using known interaction partners and by screening 160 different combinations of prey and bait proteins associated with the human androgen receptor we demonstrate that this assay allows the quantitative detection of specific protein interactions in different types of mammalian cells and under the influence of different compounds. Moreover, different strategies in respect to bait-prey combinations are presented.

**Conclusion:**

We demonstrate that the CAPPIA assay allows the quantitative detection of specific protein interactions in different types of mammalian cells and under the influence of different compounds. The high number of preys that can be tested per slide together with the flexibility to interrogate any bait of interest and the small amounts of reagents that are required makes this assay currently one of the most economical high-throughput detection assays for protein-protein interactions in mammalian cells.

## Background

Most if not all biological processes require the cooperation of pairs of proteins or the formation of large functional complexes of proteins. Therefore the analysis of protein-protein interactions, either *in vitro*, using for example protein arrays, co-immunoprecipitation or affinity chromatography, or *in vivo *by two-hybrid assays is essential for the elucidation of biological processes and/or networks.

In classical yeast or mammalian two-hybrid based assays typically two proteins of interest are ectopically expressed as fusion proteins, one with the DNA Binding Domain (DBD) of for example GAL4 or LexA and the other with a transcriptional Activating Domain (AD), such that if both proteins show any interaction, the DBD and AD are functionally linked together at the promoter, reconstituting transcriptional activity [1-3]. This causes reporters that contain GAL4- or LexA binding sequences to be transcribed.

Since two-hybrid systems are *in vivo *assays they offer advantages over *in vitro *biophysical or biochemical methods. Indeed some protein-protein interactions are too weak and/or transient to be detected *in vitro *and some of these interactions require specific post-translational modifications of the proteins and/or specific co-factors in the cellular microenvironment. For the same reasons it is advantageous to determine protein interaction networks in mammalian cells, using mammalian two-hybrid assays [4]. Until now high-throughput analyses of mammalian protein interactions were typically performed in yeast [5,6] and putative interactions were then confirmed in mammalian two-hybrid assays on a gene-by-gene scale [7,8].

We present here a novel assay for the parallel analysis of thousands of proteins for interacting partners in mammalian cells by combining cell arrays [9], with the more classical mammalian two-hybrid assay.

In this cell array protein-protein interaction assay (CAPPIA), nanoliter volumes of solutions containing a bait expression plasmid, a prey expression plasmid and a reporter plasmid complexed with transfection reagent are immobilized on glass slides in well-defined array formats. When these slides are overlayed with a monolayer of living cells, only those cells that grow on top of a particular spot of DNA will get transfected and will start to over-express specific chimeric bait and prey proteins. If these two proteins can interact with each other they will transactivate the autofluorescent reporter making that cluster of cells fluorescent while the surrounding cells remain non-fluorescent. Fluorescent cell clusters/features can then be analysed by simple fluorescence detection using common DNA array scanners or high-throughput microscopy, without the need for further manipulation of the slides such as immunofluorescence staining or enzyme-based detection.

Using known interacting proteins we demonstrate the specific and quantitative detection of protein-protein interactions on cell arrays in different mammalian cell lines. Furthermore, screening of 160 different combinations of prey and bait proteins including different domains of the human androgen receptor reveals that this assay is well suited for the detection of hormone-dependent protein interactions. The physiological significance of this interaction on cell arrays is further underscored by showing the dose response of this interaction to androgenic compounds as well as to antagonistic reagents.

Finally we demonstrate that the possible combinatorial screens to detect protein interactions can be increased by combining slides that only carry a prey library with cell lines that carry a stable or transient bait construct. This flexibility, together with the high capacity of the cell arrays and the small amounts of reagents that are required, makes this assay currently one of the most economical high-throughput detection assays for protein-protein interactions in mammalian cells.

## Results

### Development of a cell array based method for the detection of protein-protein interactions in various mammalian cells

**Figure 1:**
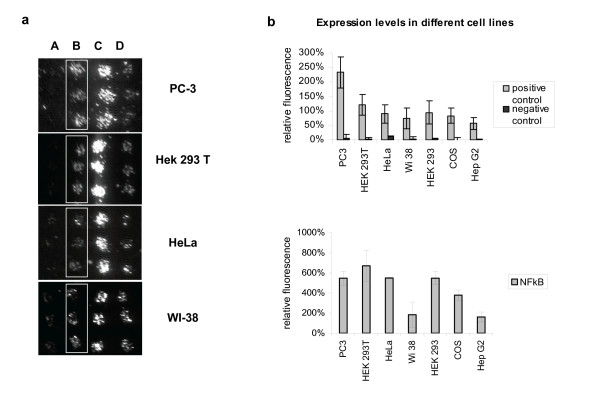
**Cell array based PPI screens in different cell lines**. **1a**: Transfection efficiency and specific protein-protein interaction in different cell lines is demonstrated using the Gal4-pZsGreen reporter and plasmids coding for the known interacting p53 (pBD-p53) and SV40-T (pAD-SV40T) hybrid proteins (boxed, line B). As negative control (line A) Gal4-pZsGreen reporter was co-transfected with the non-interacting proteins p53 (pBD-p53) and TRAF (pAD-TRAF). The pBD-NF-κB control plasmid expressing the GAL4 DNA-binding domain fused to the transcription activation domain of NF-κB is used as a positive control to monitor transfection efficiency and reporter performance (line C). A construct expressing EGFP under control of a CMV promoter (line D) is typically printed as a frame at the periphery of the arrays in order to locate the arrayed features. Fig. 1a shows example images of different adherent cell lines transfected using identical microarray slides. **1b**: Transfection efficiency as reflected by the level of NF-κB induced reporter expression. Comparable results were obtained for PC-3, HEK293 and HeLa. Data shown are from representative experiments and represent mean fluorescence of 6 features per sample, collected from triplicate spots on two identical slides.

Because cell arrays allow the simultaneous transfection and analysis of high numbers of different cDNA constructs in adherent mammalian cells [9-13] they offer the possibility to analyse thousands of proteins for interacting partners when combined with the mammalian two-hybrid assay. To improve the ease and speed of detection of protein-protein interactions (PPI) on cell arrays we created an autofluorescence-based and GAL4-driven reporter plasmid, Gal4-pZsGreen. Similarly to other two-hybrid systems, expression of this reporter gene will only occur if two chimeric proteins, a bait fused to the GAL4 DNA-binding domain and a prey fused to an activation domain, are co-expressed and subsequently form a complex with transcriptional activity. Fluorescence detection is performed using common DNA array scanners or high-throughput microscopy, eliminating the need for further manipulation of the slides. This is demonstrated in Figure 1, where microarrays were made by printing the Gal4-pZsGreen reporter mixed with pBD-p53 and pAD-SV40T, coding for two interacting proteins or mixed with pBD-p53 and pAD-TRAF, coding for two non-interacting proteins. Figure 1a, shows example images of different adherent cell lines transfected using identical microarray slides. The resulting fluorescent signals, shown in Figure 1b, were quantified as described in "Methods". Using these plasmids we show the efficient transfection and quantitative detection of PPI in six phenotypically different cell lines. Interestingly, sample and slide preparation were the same for all cell lines tested and only the number of cells and time of transfection had to be optimized for each cell type.

The observation that specific protein-protein interactions can readily be analyzed in different cell lines is of particular interest for interactions that are dependent on cell-specific posttranslational modifications of expressed proteins or that depend on cell-specific co-factors. A cell type dependent difference in PPI-induced reporter expression is already evident for p53 and SV40T proteins that induced higher reporter levels when expressed in PC-3 as compared to HEK293T cells, even though both cell lines can be transfected with similar efficiencies, as reflected by the level of NF-κB-induced reporter expression in these cell lines.

### Application of CAPPIA for screening of hormone-dependent protein interactions

**Figure 2:**
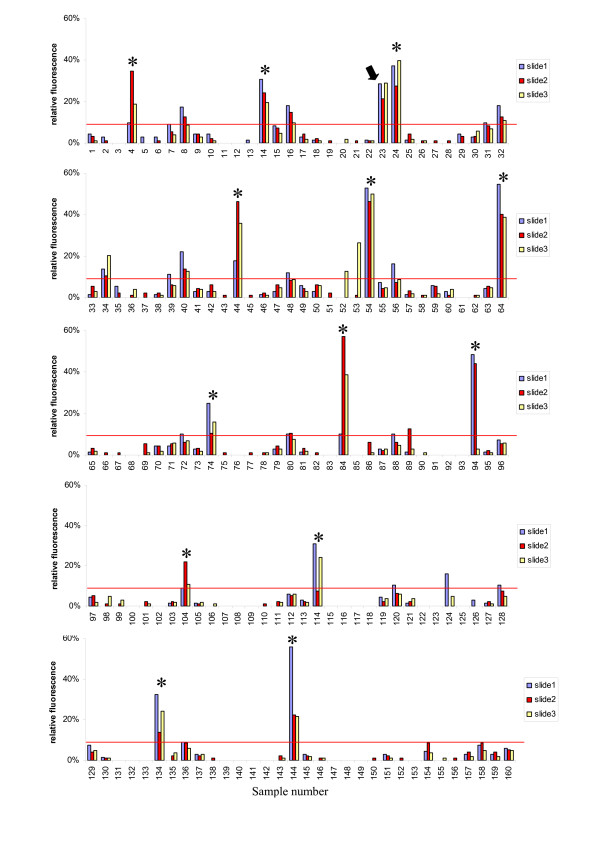
**Application of CAPPIA for the detection of hormone-dependent interactions**. Triplicate slides each carrying 208 features, including all possible combinations of the 16 prey and 10 bait proteins listed in Additional file 1 and sets of positive (p53+SV40T) and negative (p53+TRAF, single baits and preys) controls were reverse transfected with Hek293T cells in the presence of 10 nM R1881 for 3 days. Each prey-bait combination was printed together with the autofluorescent reporter as single spots per slide with a spot to spot distance of 1.5 mm. Following transfection the fluorescence signals of all 624 features were collected and processed as described in methods. The normalised fluorescence signals obtained from the 480 different bait-prey features (10 baits × 16 preys × 3 replicate slides) are shown separately for the different slides. Sample numbers correspond to the combination of bait and prey as summarized in Additional file 1. The cutoff value (indicated as red line) resembles the level of reporter expression obtained with the non-interacting control proteins p53+TRAF. This cross-screening using CAPPIA immediately identifies so-called bait-or prey-unspecific false positives as is exemplified by bait B504 (marked with a star). A specific interaction was identified between B487 and P506 corresponding to the AR-LBD and AR-NTD (sample number 23, marked with an arrow).

Having demonstrated the specificity and efficiency of CAPPIA, we next explored whether this method allows the large scale screening for hormone-dependent interactions. Steroid hormone receptors such as the androgen receptor (AR) are ligand-dependent transcription factors that control a variety of essential physiologic and developmental processes in humans [14-17]. Here, in the presence of 10 nM of the synthetic androgenic ligand R1881, we analysed the interaction of 10 individual baits with 16 different preys coding for proteins or protein domains potentially associated with nuclear receptor function (Additional file 1). Each slide contained all 160 different prey-bait combinations as single features in addition to a series of positive and negative controls to localise the array and monitor transfection efficiency as well as background fluorescence. In total each slide contained 208 spots, and three identical slides were transfected to obtain independent replicate data points for each prey-bait combination. Fluorescence signals of all 624 features were collected and processed as described in methods. The 480 data points representing reporter expression induced by the different prey-bait combinations are summarized in Figure 2. Bait B504 can immediately be recognized as being a so-called false positive bait since it activates reporter expression with multiple unrelated preys. Such false positives induce reporter expression either by unspecific binding or, as is the case for the fusion protein (AR-NTD) of bait B504, by conferring trans-activating activity to the GAL4 DNA binding domain of the bait [18]. More importantly, a specific and androgen-dependent interaction was identified between bait B487, the ligand binding domain of the AR (AR-LBD), and prey P506, the N-terminal domain of the AR (AR-NTD). This interaction was confirmed in a second CAPPIA experiment (Additional file 2).

These data are consistent with previously published androgen-dependent intra-molecular interactions between AR-LBD and AR-NTD domains [18-20].

**Figure 3:**
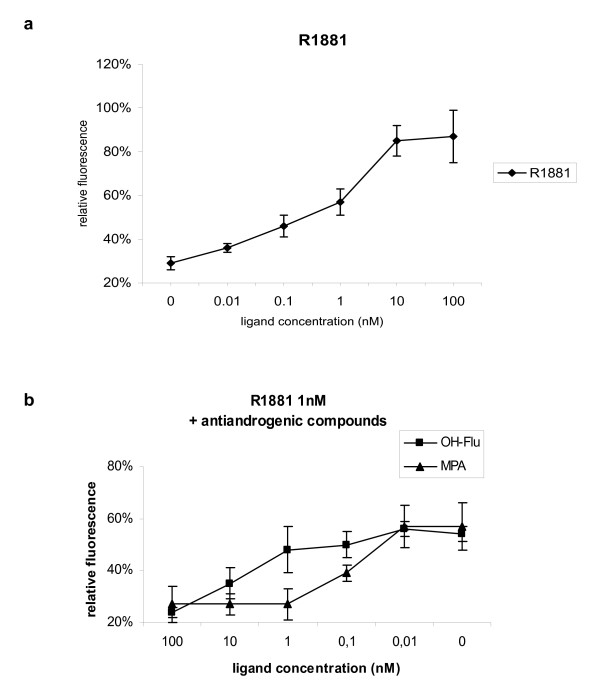
**Dose response of AR-LBD and AR-NTD interactions to androgenic and anti-androgenic compounds**. AR-LBD and AR-NTD interaction was analysed on cell arrays in the presence of different concentrations of agonist and antagonists. **3a**: Dose-dependent induction of AR-LBD and AR-NTD interaction by the synthetic agonist R1881, showing a maximal response from 10 nM onwards. **3b**: Dose-dependent inhibition of R1881 (10 nM) induced AR-LBD and AR-NTD interaction by two antagonists, medroxyprogesterone acetate (MPA) and hydroxyflutamide (OH-Flu). Quantitative analysis of this inhibition reflects the stronger antagonistic potency of MPA which can achieve maximum inhibition at a concentration of 1 nM as compared to OH-Flu, of which more than 10 nM is required to obtain the same level of inhibition. Data shown represents the mean fluorescence of 6 features per sample, collected from triplicate spots on two identical slides.

To evaluate the resolution of CAPPIA and to obtain additional evidence for the physiological significance of the AR-LBD and AR-NTD interaction using cell arrays, a dose-response curve was determined. We observed a dose-dependent induction of reporter expression in the presence of the synthetic agonist R1881 with a maximal response from 10 nM onwards (Figure 3a), in accordance with previous assays involving normal transfection of both domains and detection of GAL4-induced luciferase activity [18]. In addition we could reiterate the dose dependent inhibitory effects of two antagonists, medroxyprogesterone acetate (MPA) and hydroxyflutamide (OH-Flu) on R1881-induced AR-LBD and AR-NTD interaction on cell arrays (Figure 3b). Importantly the resolution of CAPPIA allowed the detection of quantitative differences in antagonist activity. Indeed, the R1881-induced interaction was inhibited with 1 nM MPA, whereas almost 100 nM OH-Flu was required to achieve the same level of inhibition. Similar differences in inhibitory potency between MPA and OH-Flu were also observed after normal transient transfection of both AR domains in CHO cells [21].

Taken together these experiments clearly demonstrate that cell arrays provide a functional readout to identify and monitor PPI under different physiological conditions. Besides interrogating PPI in different cellular contexts, cell arrays can also be used to screen for ligand-dependent protein-protein interactions and to quantify the dose response of these to various compounds.

### PR-stable bait and PR-trans bait cell arrays

In order to increase the possible combinatorial screens for protein interactions and hence further improve the high-throughput application of CAPPIA, we printed alternative slides on which the bait was omitted. Each spot on these so-called prey-reporter- (PR-) slides contains only the reporter and one of the prey constructs. To screen for interacting partners the bait is then introduced into the cells before adding them to the prey-reporter-arrays. This is either done using stably transfected cell lines (PR-stable-bait assay) or by transiently transfecting cells with the bait shortly before adding them to the arrays (PR-trans-bait assay). In both cases all the cells on these transfected slides express the bait. However, only cells that grow on top of a spot, with a prey that can interact with that bait, become fluorescent.

**Figure 4:**
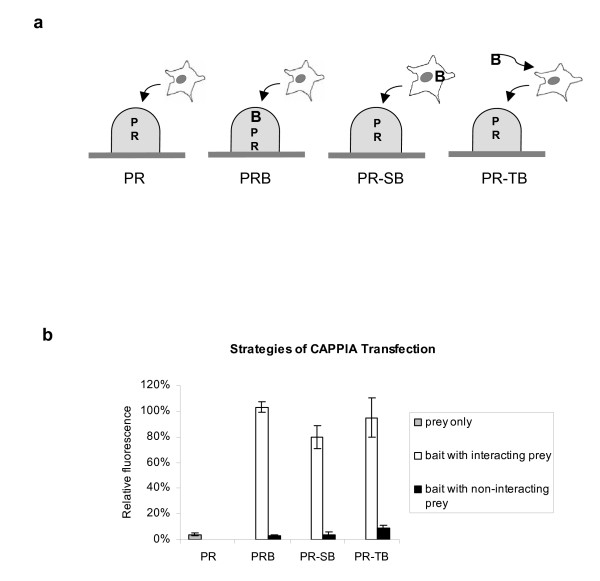
**PR-stable and PR-trans bait cell arrays**. **4a**: Schematic representation of different transfection strategies tested in CAPPIA. On Prey-Reporter-Bait (PRB) slides, bait and prey expression plasmids AR-LBD and AR-NTD respectively and the reporter plasmid complexed with transfection reagent were immobilized together in array format. In contrast, on PR slides, each spot contained only the reporter and the AR-NTD prey construct. For Prey-Reporter-stable-bait (PR-SB) experiments a HEK293 cell line with a stable integration of the pBD-LBD plasmid was generated and grown on top of the PR slides. For Prey-Reporter-transient-bait (PR-TB) experiments, suspensions of HEK293T cells were incubated with pBD-LBD plasmid complexed with transfection reagent 5 minutes before adding them to the PR-slides. Finally PRB slides were incubated with non-treated HEK293T cells as described before. All cells were cultured on the slides for 3 days in the presence of 10 nM R1881. **4b**: When the data are normalised for differences in transfection efficiencies the results show that all three strategies result in a comparable specific trans-activation of reporter expression following AR-LBD and AR-NTD interaction. Data shown represents the mean fluorescence of 6 features per sample, collected from triplicate spots on two identical slides.

We used the hormone-dependent AR-LBD and AR-NTD interaction to compare the "PR-stable-bait" and "PR-trans-bait" strategies with the results obtained with the original PRB slides described earlier. A schematic representation of the three strategies with the corresponding results is shown in Figure 4. After normalisation of the data so as to correct for differences in transfection efficiencies the results show that all three strategies result in a comparable trans-activation of Gal4-pZsGreen as a result of AR-LBD and AR-NTD interaction.

Although PRB slides offer a cost-effective and robust platform for simultaneous comparison of large number of interactions in different cell lines and under different culture conditions (time of culture, addition of agonists or antagonists), the PR slides are more suitable for the large-scale screening of novel bait-prey interactions. As shown in Additional file 3, the spot to spot distance on CAPPIA slides can easily be reduced to 1 mm. By printing each sample as one feature per slide and using a spot distance of 1 mm, up to 900 different preys can be printed on one slide. Replicate PR-slides can then be screened with any bait of interest, further increasing the high-throughput application of CAPPIA. In addition PR slides can be printed in large batches and can be stored for at least 6 months at 4°C and even longer at -80°C (data not shown), thus increasing the cost effectiveness of CAPPIA. The slides can be shipped on ice, allowing any research group with access to standard culture facilities to interrogate the prey library with their bait of interest.

## Discussion

We hereby present a new screening technology for the high-throughput interrogation of protein-protein interactions in mammalian cells. This cell-array-protein-protein-interaction-assay (CAPPIA) combines the advantages of mammalian two-hybrid systems with those of microarrays, a combination that is expected to save considerable time and expense. Indeed, CAPPIA slides are printed with the same robotic microarray devices used to print conventional DNA microarrays. Consequently, cell arrays require far less DNA, transfection reagents and cells as compared to assays performed in microwell plate format.

For comparison, the amount of transfection reagent required to transfect one sample in a 96 micro-well is sufficient to transfect more than 2000 samples in CAPPIA, a significant cost reduction since transfection reagents account for a major part of the expenses in these assays. Although cell arrays can theoretically carry up to 8000 features per slide [9-11,13], the actual number of features per slide is dependent on the type of application. Since the efficiency of the simultaneous transfection of three plasmids is lower than that of a single plasmid and since the reporter will only be expressed after sufficient amounts of both prey and bait proteins are expressed in the same cell there is a need to increase the number of cells per spot in CAPPIA in order to guarantee the robust detection of interacting proteins. Consequently, in order to increase the sensitivity of the assay, the features on CAPPIA slides need to be bigger and hence the slide capacity will be lower. Still, CAPPIA slides can be printed with each containing up to 900 different preys as single spots when using a spot to spot distance of 1 mm. This is equivalent to 9 standard 96 micro-well plates and data can then be collected from a number of identical slides to obtain replicate data points per sample. In addition, by pooling 3 preys per feature even more preys can be interrogated per slide. The cost-effectiveness of the assay is further increased by using an auto-fluorescent based reporter eliminating the need for immuno-fluorescence staining or enzyme-based reporter detection. In contrast to microwell-based assays no extensive liquid handling infrastructure is required once the slides have been printed. In addition PR or PRB slides can be printed in large batches and stored frozen without losing their efficiency, thus increasing the flexibility and cost effectiveness of CAPPIA. Finally, PR slides can be screened with any bait of interest simply by using standard transfection and cell culture methods, available to many research groups.

Compared to the more common high-throughput yeast two-hybrid assay, CAPPIA offers the advantage of testing mammalian PPI within a cellular context that more closely mimics the native protein environment. As a consequence the rate of false negative interactions, i.e. true interactions that can not be detected due to the inappropriate folding and/or post-translational processing of the proteins should be lower using CAPPIA, especially since phenotypically different cell types can be used. As with DNA microarrays, PR-slides interrogated with different baits allow a comparison between multiple identical assays. As such preys or baits that are found multiple times with unrelated proteins, so-called bait- or prey-unspecific false positives [22,23] can be distinguished without need for further experimentation. High-throughput yeast two-hybrid assays also require extensive liquid handling infrastructures [5,24] making the assays less accessible for many research groups.

Previously, Suzuki et al [25] described a PCR-mediated rapid sample preparation and a high-throughput assay system based on the mammalian two-hybrid method. Although the method they designed allows for the rapid preparation of high numbers of bait and prey samples, the actual two-hybrid assays are performed in microwells and require semiautomatic multiple dispensers as well as multiple reagents for downstream enzymatic detection of interacting proteins. CAPPIA, on the other hand allows the quantitative analysis of interactions comparable to assays performed in microwells, while offering the advantage of being economical and accessible to research groups equipped with basic cell culture infrastructures. Currently, large bait-prey libraries for use in CAPPIA are being generated using Gateway (Invitrogen) compatible destination pCMV-AD and pCMV-BD vectors and panels of pENTRY clones. Since CAPPIA is based on the GAL4-two-hybrid system that is already used by many research groups, existing baits and preys can easily be included in the assays. In particular, the trans-bait version of CAPPIA readily allows available bait constructs to be screened against large number of preys.

## Conclusion

Taken together, the high capacity of the cell arrays, the flexibility to interrogate any bait of interest and the small amounts of reagents that are required make CAPPIA currently one of the most economical high-throughput detection assays for protein-protein interactions in mammalian cells. Microarray-based systems such as the one presented here should lead to the acceleration of the deciphering of functional protein complexes. In addition CAPPIA could also be an economical alternative for the identification of potential drug targets by combining screens for small molecule ligands that can disrupt or modulate interactions with selected prey-bait combinations expressed in array format inside 96 micro-wells [26].

## Methods

### Cell lines

WI-38, SV40-transformed fibroblasts; HeLa, epithelial cervical carcinoma; COS 7 African green monkey kidney cells; HEK293 embryonic kidney cells and its variant HEK293T were from ATTC. PC-3 human prostate cancer cells were from the German Collection of Microorganisms and Cell Cultures (Braunschweig); the HepG2, hepatocellular carcinoma was a kind gift from Dr. S. Sperling (MPI-MG, Berlin). Cells were cultured at 37°C, 5% CO_2 _in D-MEM (GIBCO) with 10% fetal bovine serum, penicillin/streptomycin and 1% L-glutamine.

HEK293-LBD cells were created by transfecting HEK293 with pBD-LBD and selecting for Neomycine-resistant transformants. HEK293 cells instead of HEK293T cells were used for these experiments since the latter is already Neomycine resistant.

### Plasmids

Expression bait (pCMV-BD) and prey (pCMV-AD) vectors used to generate proteins of interest as GAL4 DBD fusions and fusions with the transactivation domain of NF-κB, respectively were from Mammalian Two-Hybrid Assay Kit from Stratagene. The regions coding for the different proteins or protein domains, summarized in Additional file 1, were introduced into pCMV-BD and pCMV-AD using standard methods. Positive and negative control plasmids from the kit were pBD-NF-κB, pBD-p53, pAD-SV40T and pAD-TRAF.

A CMV-driven EGFP construct was generated by PCR amplification of EGFP from pIRES2-EGFP (Clontech) and TA cloning into pcDNA4/HisMax TOPO (Invitrogen).

The autofluorescent reporter Gal4-pZsGreen was created by cloning the GAL4 upstream activating sequences from pGAL/lacZ (Invitrogen) into the multicloning sites of pZsGreen1-1 (Clontech). The pCIS-CK plasmid was from Stratagene.

### Sample preparation

Samples were prepared as described using the lipid-DNA-method [9,10]. Briefly, DNA mixtures containing reporter and prey (for PR slides) or reporter, prey and bait constructs (for PRB slides) in EC buffer supplemented with 0.2 M sucrose were treated for 5 minutes at RT with 1.5 μl Enhancer (Effectene, QIAGEN). Subsequently, 7 μl Effectene (QIAGEN) was added and 10 minutes later an equal amount of 0.1% gelatine (SIGMA) solution in water was added. Samples were used immediately or stored for several days at 4°C. Where required pCIS-CK was used to obtain a final DNA concentration of 50 ng/μl.

### Preparation of arrayed slides

Arrays were printed using a sciFlexArrayer non-contact piezodispensing system (Scienion AG) with 70 μm nozzle. Features were printed with a spot-to-spot distance of 1.5 mm using about 6 nl sample per feature. Slides used were home made poly-L-lysine (SIGMA) coated plain microscope glass slides.

For small number of samples, manual spotting is a useful alternative to the use of automated array robots. About 10 nl sample was manually deposited per spot using long tips (PreCision safe seal tips, 10 μl, Biozym) by gently touching the filled tip on top of the slide surface. Slides were positioned on a frame allowing the arrayed dispensing of spots at 1.5 mm intervals.

Printed slides were dried for a minimum of 1 hour before transfection or were stored for longer times desiccated at 4°C or -20°C.

### Transfection of cell arrays

For HEK293T, HEK293, HEK293-LBD, PC-3, WI-38 and HepG2, 1 × 10^7 ^cells were seeded in a 60 cm^2 ^cell culture plate one day before transfection. For HeLa and Cos7, 5 × 10^6 ^cells were pre-cultured in a 145 cm^2 ^plate. On the day of transfection cells were seeded at 3.5 × 10^6 ^(HEK293T, HEK293, HEK293-LBD, PC-3, Cos7, HepG2 and WI-38) or 1 × 10^6 ^(HeLa) cells per slide in 8 ml complete media in quadriperm (Vivascience) dishes. For hormone-dependent interactions the medium was supplemented with R1881 (Methyltrienolone, Perkin Elmer) and Medroxyprogesterone acetate (MPA, Schering AG) or hydroxyflutamide (OH-Flu, Schering AG) at the indicated concentrations. After 48 or 72 hours the slides were washed with PBS and fixed with PBS/4M sucrose/3.7% formaldehyde solution for 30 min. After washing in PBS, slides were mounted with Fluoromount-G (Southern Biotech) and stored in the dark at 4°C until analysis.

### Signal detection and analysis

Fluorescent signals were detected using a DNA microarray reader (BIOCCD Image Reader, PE Applied Biosystems) with excitation 470/30 and emission 510/20 filters for EGFP and ZsGreen. The entire slide was uniformly scanned with same focus and exposure time settings. Pictures were handled by AxioVision LE (ZEISS) and image densitometry analysis was done with AlphaEase FC Stand Alone Software, version 4.0.0 (Alpha Inotech). Using the "Spot Denso" tool integrated in the software a "Spot denso overlay" was generated, stored and loaded for each new scanned slide that was printed with the same feature size and pitch parameters. Using this overlay the relative grey value of each feature of the slide is automatically analysed and exported as an excel file for further processing. Background fluorescence of cells is subtracted before data is exported. Data was collected from identical slides to achieve a number of replicate data points per sample as indicated. Exposure times were chosen so that the weaker, experimental signals are within linear range. No selection markers were used to monitor transfection of individual plasmids. Signals were normalized for transfection efficiency relative to the level of EGFP expression on the same slide and the mean value from replicate spots was calculated for each sample.

## Abbreviations

PPI, protein-protein interaction; DBD, DNA binding domain; AD activating domain; CAPPIA, cell array protein-protein interaction assay; PRB, prey, reporter, bait; PR prey, reporter; R1881, methyltrienolone; MPA, medroxyprogesterone acetate; OH-Flu, hydroxyflutamide; AR, androgen receptor; AR-LBD, androgen receptor ligand binding domain; AR-NTD, androgen receptor N-terminal domain

## Authors' contributions

A.F. optimized the cell array protocol, performed CAPPIA studies and was pivotal in writing the manuscript; L.N. and F.W. were instrumental in optimizations and production of DNA arrays for reverse transfections; B.H. was involved in androgen receptor studies; Y.H.H. was involved in establishment of the transfected-cell array protocol; S.T. assisted in maintenance of cell cultures; H.L. was involved in conceptualization of the study; M.J. and D.V. supervised the study and were involved in the conceptualization and writing.

All authors read and approved the final manuscript.

## Supplementary Material

Additional file 1 Supplemental table 1. Sample numbers representing different combinations of bait and prey proteins used to screen for hormone dependent interactions. The table describes spotting configurations of expression plasmid constructs used in the study. Click here for file

Additional file 2 Supplemental figure 1. Re-screening of selected bait and prey constructs. Bait B487 coding for AR-LBD was re-analysed with the 16 different preys summarized in Table 1 of the manuscript. For this purpose triplicate spots of each prey-reporter-bait combination (represented by a sample number as described in supplementary Table 1), positive control (a) p53+SV40T and negative controls (b) p53+TRAF, (c): SV40T and (d) TRAF were printed and used to reverse transfect Hek293T cells in the presence of 10 nM R1881 for three days. Data was collected from 2 replicate slides to obtain the mean fluorescence value of 6 replicate spots. Also in this experiment AR-LBD was found to specifically interact with AR-NTD, the N-terminal domain of the AR but not with any of the other preys. Fig. 1a: BIOCCD scanner image of a representative slide. Fig. 1b: Relative fluorescence signal obtained for the different bait-prey combinations. Click here for file

Additional file 3 Supplemental figure 2. Determination of optimal spot to spot distance. Using the SciFlexarray printer system a series of samples containing a CMV-driven EGFP construct but with different DNA and or gelatine concentrations were printed in sub-arrays with different spot to spot distances. Slides were transfected with HEK293T cells during 3 days. Clusters of fluorescent cells transfected with the same sample, but distributed with different spot to spot distances are highlighted on the array using white boxes. It is clear from this picture that a distance of 1 mm is the optimal distance to obtain high-density slides while maintaining enough space between the spots. Same array parameters can also be used for transfection of bigger cells such as HeLa since the size of the cells does not significantly change the size of the transfected spots (data not shown). The main consequence of using bigger cells is that fewer cells are transfected per spot as compared to smaller cells. Click here for file
