# Supplementary material for: High-throughput mammalian two-hybrid screening for protein-protein interactions using transfected cell arrays
Source: BMC Genomics. 2008 Feb 6;9:68. doi: 10.1186/1471-2164-9-68 (PMC2254387; doi:10.1186/1471-2164-9-68)
Supplement: Additional file 1 — Supplemental table 1. Sample numbers representing different combinations of bait and prey proteins used to screen for hormone dependent interactions. The table describes spotting configurations of expression plasmid constructs used in the study. [file 1471-2164-9-68-S1.pdf]

Supplementary Table 1. Sample numbers representing different combinations of bait and prey proteins used to screen for hormone dependent interactions.

Bait / Prey	Hinge region of AR	DBD of AR	LBD of AR	NTD of AR	OTEX	SH3	NTD of GR	ALIEN domain	NcoR domain	SMRT domain
Hinge region of AR	1	2	3	4	5	6	7	8	9	10
DBD of AR	11	12	13	14	15	16	17	18	19	20
NTD of AR	21	22	23	24	25	26	27	28	29	30
LBD of AR	31	32	33	34	35	36	37	38	39	40
Pea3 (full-length)	41	42	43	44	45	46	47	48	49	50
Pea3 N-terminal domain	51	52	53	54	55	56	57	58	59	60
Pea3 middle domain	61	62	63	64	65	66	67	68	69	70
Pea3 C-terminal domain	71	72	73	74	75	76	77	78	79	80
OTEX	81	82	83	84	85	86	87	88	89	90
Menin (aa 1-455)	91	92	93	94	95	96	97	98	99	100
Menin (aa 224-455)	101	102	103	104	105	106	107	108	109	110
Menin (aa 456-615)	111	112	113	114	115	116	117	118	119	120
Menin (aa 224-615)	121	122	123	124	125	126	127	128	129	130
Menin (aa 1-223	131	132	133	134	135	136	137	138	139	140
ALIEN domain	141	142	143	144	145	146	147	148	149	150
SMRT domain	151	152	153	154	155	156	157	158	159	160

Prey and bait constructs were generated by inserting the indicated coding region in frame with the NF-κB activation domain of pCMV-AD and the GAL4 DNA binding domain of pCMV-BD, respectively. The different preys and baits include different domains of the human androgen receptor and proteins potentially associated with nuclear receptor function. Each sample number in the table corresponds to a particular combination of bait and prey identified respectively by the name in the first row and first column of the table.
