# Supplementary figures and images for: High-throughput mammalian two-hybrid screening for protein-protein interactions using transfected cell arrays
Source: BMC Genomics. 2008 Feb 6;9:68. doi: 10.1186/1471-2164-9-68 (PMC2254387; doi:10.1186/1471-2164-9-68)

1a

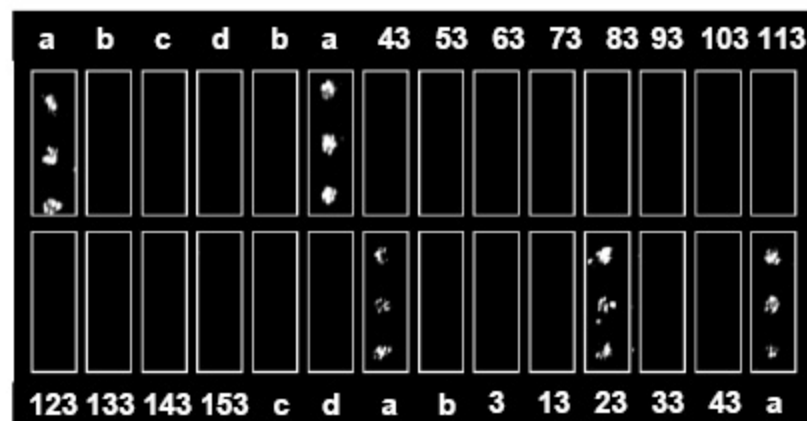

1b

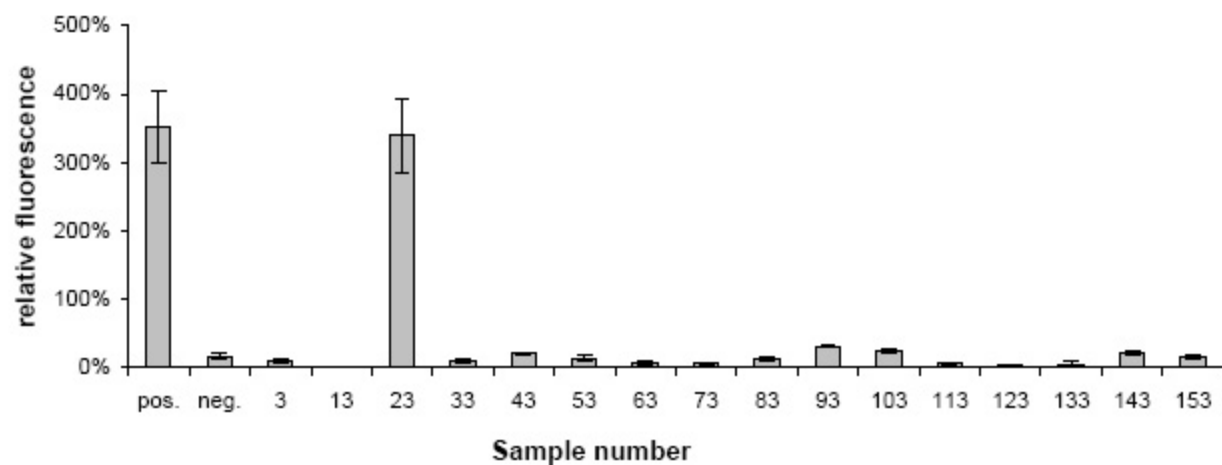

Supplement: Additional file 2 — Supplemental figure 1. Re-screening of selected bait and prey constructs. Bait B487 coding for AR-LBD was re-analysed with the 16 different preys summarized in Table 1 of the manuscript. For this purpose triplicate spots of each prey-reporter-bait combination (represented by a sample number as described in supplementary Table 1), positive control (a) p53+SV40T and negative controls (b) p53+TRAF, (c): SV40T and (d) TRAF were printed and used to reverse transfect Hek293T cells in the presence of 10 nM R1881 for three days. Data was collected from 2 replicate slides to obtain the mean fluorescence value of 6 replicate spots. Also in this experiment AR-LBD was found to specifically interact with AR-NTD, the N-terminal domain of the AR but not with any of the other preys. Fig. 1a: BIOCCD scanner image of a representative slide. Fig. 1b: Relative fluorescence signal obtained for the different bait-prey combinations. [file 1471-2164-9-68-S2.pdf]

Supplementary Figure 2

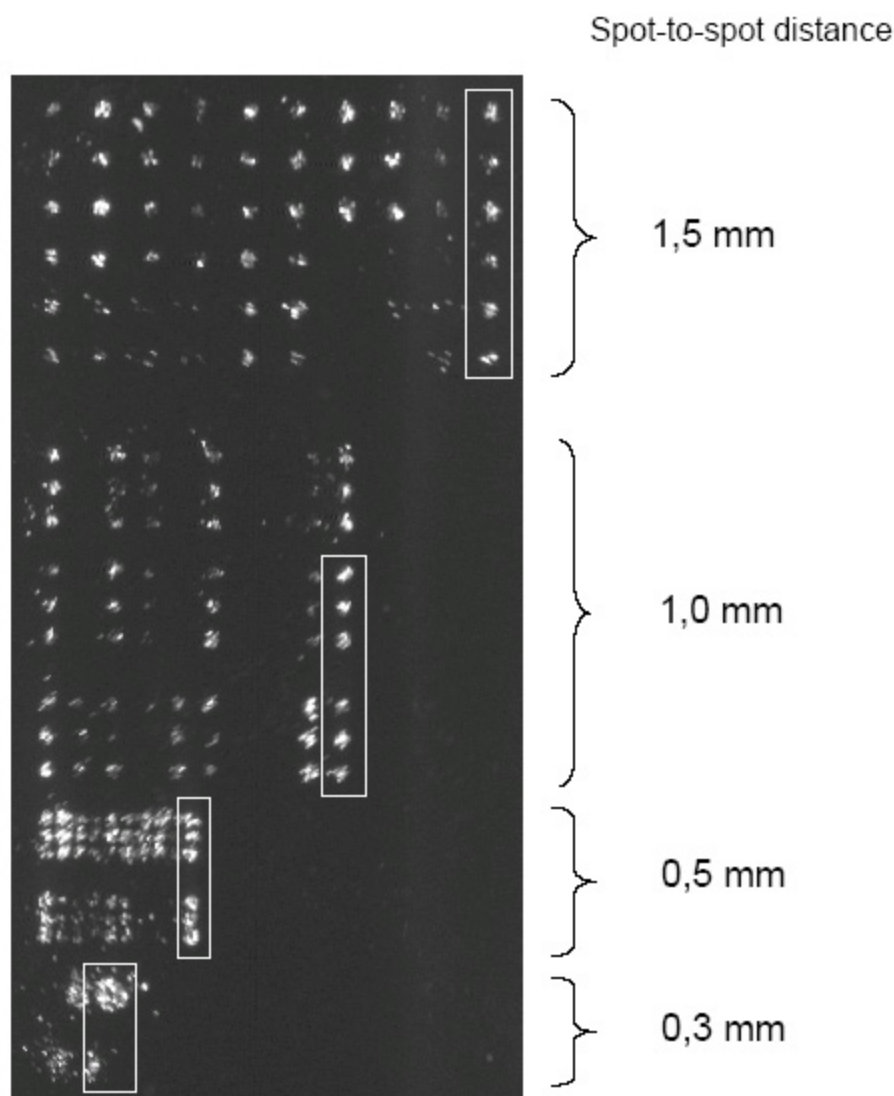

Supplement: Additional file 3 — Supplemental figure 2. Determination of optimal spot to spot distance. Using the SciFlexarray printer system a series of samples containing a CMV-driven EGFP construct but with different DNA and or gelatine concentrations were printed in sub-arrays with different spot to spot distances. Slides were transfected with HEK293T cells during 3 days. Clusters of fluorescent cells transfected with the same sample, but distributed with different spot to spot distances are highlighted on the array using white boxes. It is clear from this picture that a distance of 1 mm is the optimal distance to obtain high-density slides while maintaining enough space between the spots. Same array parameters can also be used for transfection of bigger cells such as HeLa since the size of the cells does not significantly change the size of the transfected spots (data not shown). The main consequence of using bigger cells is that fewer cells are transfected per spot as compared to smaller cells. [file 1471-2164-9-68-S3.pdf]
